# Localized peritoneal epithelioid clear cell subtype mesothelioma: a case report and literature review

**DOI:** 10.3389/fonc.2026.1790964

**Published:** 2026-05-14

**Authors:** Bin Huang, Hongsheng Liu, Haiyan Zhou

**Affiliations:** 1 Department of Pathology, The First People’s Hospital of Xiaoshan District, Hangzhou, China; 2 Department of General Practice, Chengxiang Sub-district Community Health Service Center of Xiaoshan District, Hangzhou, China

**Keywords:** peritoneal, localized, clear cell subtype, mesothelioma, clinicopathology

## Abstract

Epithelioid mesothelioma with a clear cell morphology is rare and has only been described in a few case reports. The diagnosis of epithelioid clear cell mesothelioma is challenging and often requires extensive immunohistochemical staining. Here, we report a case of epithelioid mesothelioma, clear cell variant, occurring in the peritoneum of a 76-year-old male. Computed Tomography(CT)imaging revealed a localized irregular intra-abdominal mass measuring approximately 6.0×5.0cm. Combined with histomorphology and immunophenotype analysis, the patient was diagnosed with localized epithelioid clear cell mesothelioma. There was no recurrence or metastasis after 24 months of follow-up. This case enriches the clinical and molecular data of localized peritoneal epithelioid clear cell mesothelioma, and provides insights into its diagnosis, differential diagnosis, and treatment.

## Introduction

Malignant mesothelioma (MM) is a rare and difficult-to-treat primary tumor, accounting for approximately 0.2% of all malignant tumors. It commonly occurs in the pleura, while malignant peritoneal mesothelioma (MPM) is relatively rare. Malignant mesothelioma is characterized by a variety of histological and cytomorphological patterns. It is classified into diffuse and localized types, with diffuse malignant mesothelioma generally divided into three types: epithelioid, sarcomatoid, and biphasic mixed type. Among these, the epithelioid type is the most common, while the biphasic type is less common (1). Although epithelioid mesothelioma is the most common subtype, it also presents many uncommon histological variations. These include adenomatoid tumors and tumors composed of small cells or mucin-positive cells, as well as variants such as trabecular, tubular, cord-like, fascicular, small cell, vacuolated, and clear cell types (2, 3). Clear cell mesothelioma, also known as glycogen-rich mesothelioma or foamy mesothelioma, is a very rare subtype with only a few reported cases. It can originate from the pleura or peritoneum. Due to the presence of various tumors with similar morphological features, diagnosing clear cell peritoneal epithelioid mesothelioma can be challenging (4). Among the various auxiliary techniques available for the differential diagnosis of epithelioid mesothelioma, immunohistochemistry is considered the most practical method. Due to its non-specific clinical manifestations and overlapping morphological features with other tumors (e.g., gastrointestinal stromal tumor, metastatic adenocarcinoma), misdiagnosis is common. Herein, we report a case of localized peritoneal epithelioid clear cell subtype mesothelioma with detailed clinicopathological, immunohistochemical, and genetic data, aiming to improve the diagnostic accuracy and clinical management of this rare disease.

## Case report

The patient is a 76-year-old male who was admitted to The First People’s Hospital of Xiaoshan District Hangzhou, due to upper abdominal distension lasting more than 10 days. The patient began experiencing discomfort in the upper abdomen without any obvious cause over 10 days prior, with no significant abdominal pain but noticeable bloating and belching. There was no nausea or vomiting, but the patient had a poor appetite and bowel movements occurred once every 3–4 days, with hard stools and no signs of blood. The patient had no history of occupational exposure such as asbestos exposure. There was no ascites on admission, and ascites cytology was not performed.

Physical examination revealed that the patient was conscious and in fair spirits. The abdomen was flat and soft, with no visible gastrointestinal peristalsis or waves, no tenderness or rebound pain, and no palpable masses. The liver and spleen were not palpable below the ribs, and there was no percussion pain in the liver and kidney areas. Murphy’s sign was negative, shifting dullness was absent, and bowel sounds were normal at 3–5 times per minute.

**Figure 1:**
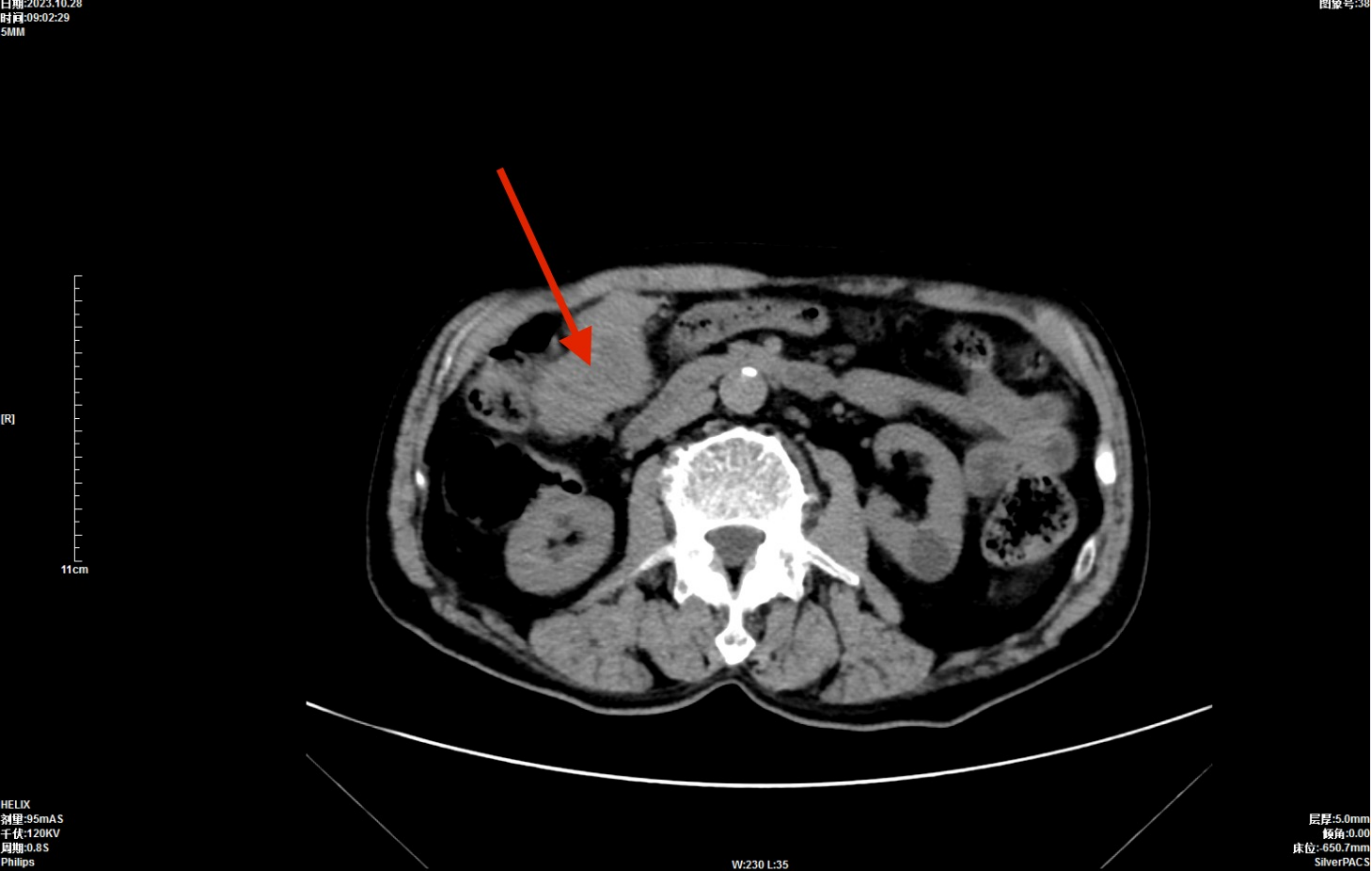
Enhanced CT showing an irregular mass shadow (arrow) in the right mid-upper abdomen.

Contrast-enhanced CT(Computed tomography) scan showed an irregular mass in the right upper abdomen (**Figure 1**), with heterogeneous density and measuring approximately 6.0×5.0 cm. The mass exhibited uneven enhancement after contrast administration and was closely related to the hepatic flexure of the colon, no ascites, peritoneal nodules, or enlarged lymph nodes were observed, suggesting a possible gastrointestinal stromal tumor (GIST). Serum tests indicated that carbohydrate antigen 125 (CA125), carcinoembryonic antigen (CEA), alpha-fetoprotein (AFP), and prostate-specific antigen (PSA) levels were within normal ranges. Stool routine and occult blood tests showed no abnormalities.

The patient underwent a “right hemicolectomy + adhesiolysis + ileo-transverse colon side-to-side anastomosis” under general anesthesia. During the surgery, a 7.0×5.0×4.0 cm mass with an uneven surface, well-demarcated boundaries, and a hard texture was found near the serosa of the transverse colon close to the hepatic flexure. Exploration of the rest of the colon, liver, stomach, pancreas, spleen, and retroperitoneum revealed no significant abnormalities.

Pathological examination results show that the length of the colon in the radical resection specimen of the right colon tumor is 19 cm, with a circumference of 5.5 cm. The mucosal surface is smooth, and an irregular nodular mass measuring 7.0×4.5×4.0 cm is found on the serosal surface, 6.0 cm from the distal resection margin. The cut surface of the mass is variegated and soft in texture. The length of the small intestine is 5.0 cm, with a circumference of 4.0 cm, and the mucosal surface is smooth. An appendix measuring 4.0×0.4 cm is found in the ileocecal region. The accompanying omental tissue measures 15.0×5.0×1.0 cm, with no other nodules palpated.

**Figure 2:**
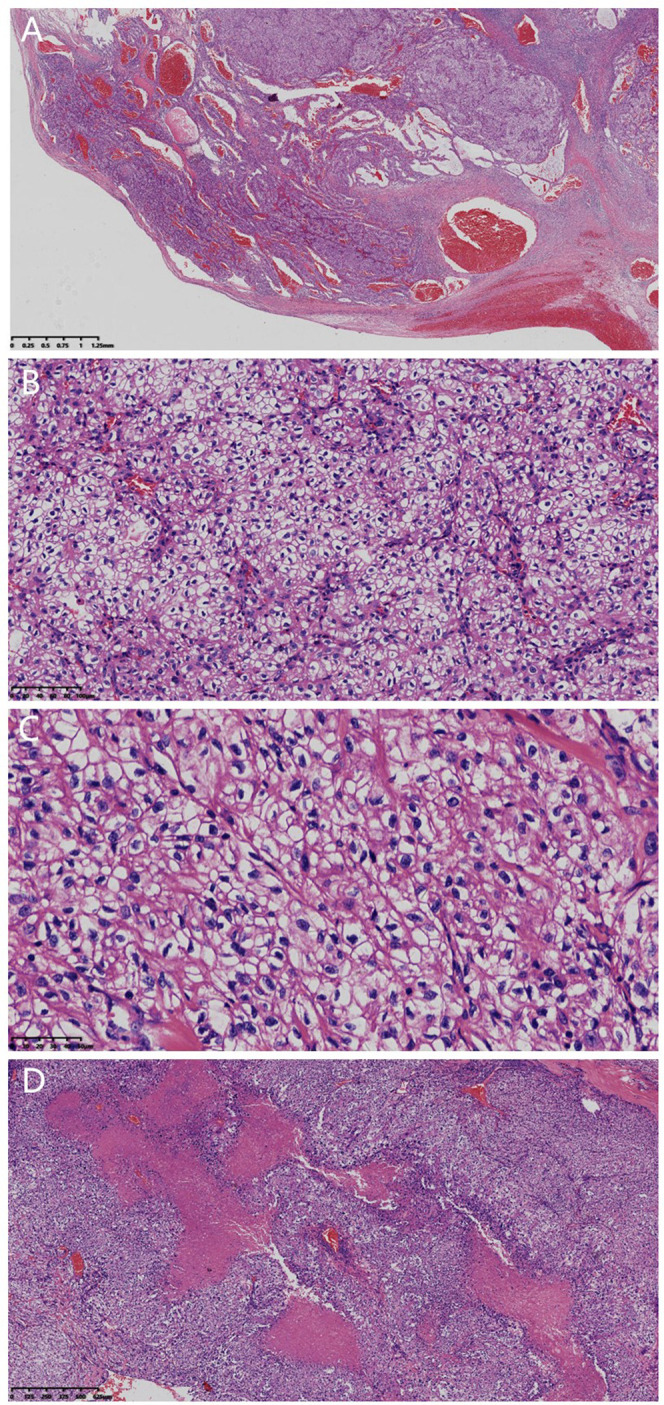
Histopathological findings (H&E staining). **(A)** Well-circumscribed tumor with predominant glandular/solid structures and dilated blood vessels (×16, scale bar=1.25 mm). **(B)** Epithelioid cells with moderate atypia and clear cytoplasm (×200, scale bar=100 μm). **(C)** Epithelioid cells had irregular nuclei with a high nuclear to cytoplasmic ratio (×400, scale bar=50 μm). **(D)** Extensive tumor necrosis (×32, scale bar=625 μm).

Hematoxylin-Eosin(HE) staining. Tissues were fixed in 10% neutral buffered formalin for 24 hours, embedded in paraffin, and sectioned into 3μm continuous slices. Microscopic examination reveals that under low magnification, the tumor boundary is well-circumscribed. The tumor parenchyma is mainly composed of epithelioid cells, presenting predominantly glandular and solid structures (**Figure 2A**), with a small amount of cystic and papillary structures. Epithelioid cells showed moderate atypia, cytoplasmic transparency (**Figures 2B, C**), variable nuclear size, irregular nuclear shape, high nucleus-cytoplasmic ratio, visible nucleolus, and rare mitosis.There are multiple areas of extensive necrosis within the tumor (**Figure 2D**). The stroma is rich in thin-walled blood vessels, accompanied by lymphocyte and plasma cell infiltration.

**Figure 3:**
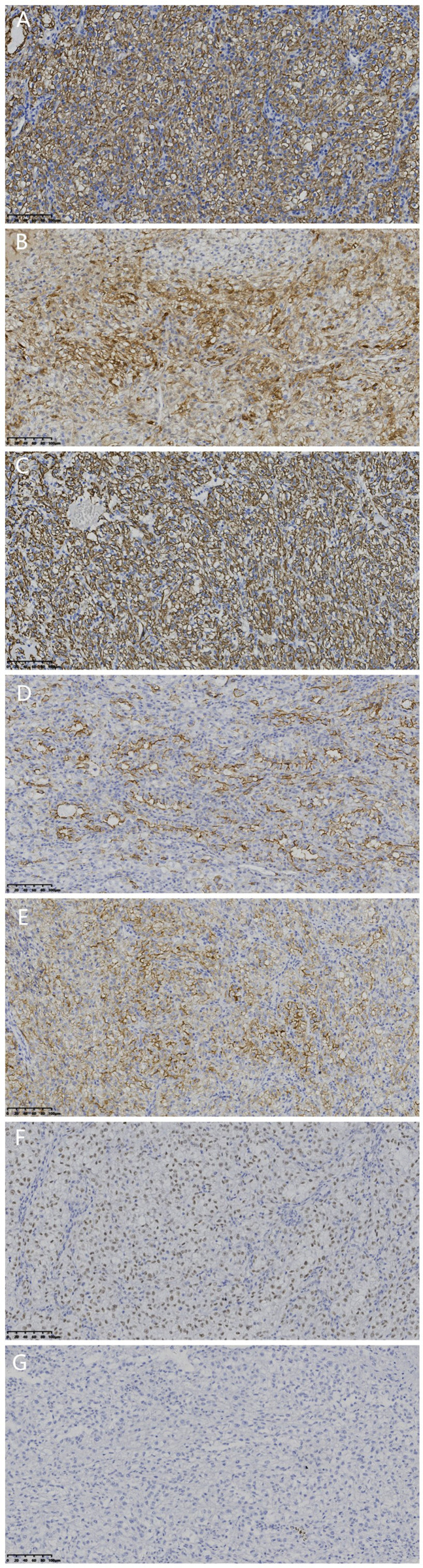
Immunohistochemical findings (×200, scale bar=100 μm for all). **(A)** Tumor cells were positive for CK. **(B)** Tumor cells were positive for calretinin. **(C)** Tumor cells were positive for desmin. D: Tumor cells were positive for D2-40. **(E)** Tumor cells were positive for HBME-1. **(F)** Tumor cells were weakly positive for WT-1. **(G)** Tumor cells were negative for BAP-1.

Immunohistochemistry (IHC) analysis was performed using an EnVision IHC kit (polymer method; cat. no. KIT-0014; Fuzhou Maixin Biotechnology Development Co., Ltd.) using primary antibodies (incubated for 50 min at 25˚C) purchased from Beijing Zhongshan Jinqiao Biological Co., Ltd. and Fuzhou Maixin Biotechnology Development Co., Ltd. The results show positivity for Cytokeratin (CK) (**Figure 3A**), Calretinin (**Figure 3B**), and Desmin (**Figure 3C**), partial positivity for D2-40 (**Figure 3D**) and Hector Battifora Mesothelial-1 (HBME-1) (**Figure 3E**), weak positivity for Wilms Tumor Protein 1 (WT-1) (**Figure 3F**), and partial positivity for Vimentin. However, BRCA1 Associated Protein 1 (BAP1) (**Figure 3G**), Cytokeratin 5/6 (CK5/6), Paired Box 8 (PAX-8), Renal, α-Methylacyl-CoA Racemase (p504S), CD34, CD117, DOG1, GATA Binding Protein 3 (GATA3), Hepatocyte, S-100 Protein (S-100), Human Melanoma Black 45 (HMB45), Melan-A, Cytokeratin 20 (CK20), Caudal Type Homeobox Transcription Factor 2 (CDX-2), Myogenic Differentiation 1 (Myo-D1), Myoglobin, Caldesmon, and Smooth Muscle Actin (SMA) are all negative. The Ki-67 Proliferation Index (Ki-67) is 20%.

We also performed tumor genetic testing for the patient (next-generation sequencing, NGS). The solid tumor 128-gene panel testing was conducted by Shanghai Keyi Lianchuang Medical Laboratory Co., Ltd (China). The results revealed: Anaplastic Lymphoma Kinase (ALK) exon 20 (c.C3358T) mutation with a variant allele frequency of 8.77%, MutS Homolog 2 (MSH2) exon 12 (c.A1837T) mutation with a frequency of 3.25%, and Ataxia Telangiectasia Mutated (ATM) exon 18 (c.C2812T) mutation with a frequency of 5.23%. The clinical significance of these specific mutations remains unknown. Microsatellite instability (MSI) analysis showed MSI-L (microsatellite instability-low), suggesting potential insensitivity to immunotherapy.

Finally, the patient was diagnosed with localized peritoneal epithelioid clear cell subtype mesothelioma (right mesocolon). The tumor was surgically resected, and the patient received no postoperative radiotherapy or chemotherapy. During 24 months of follow-up, the patient maintained disease-free survival.

## Discussion

This case involves a 76-year-old male patient presenting with non-specific epigastric distension as the primary clinical manifestation. Imaging studies revealed a mass adjacent to the hepatic flexure of the colon, with subsequent histopathological, and immunohistochemical confirmation of localized epithelioid clear cell subtype mesothelioma of the peritoneum (right mesocolon). The case demonstrates distinctive features in clinical diagnosis, pathological characteristics, and therapeutic management strategies, warranting in-depth clinical discussion.

The patient’s clinical presentation primarily includes abdominal distension and constipation, lacking specific tumor-related symptoms (such as abdominal pain, bloody stools, weight loss, etc.). There was no ascites on admission, and no peritoneal fluid cytology specimens were obtained. Imaging studies indicate a mass in the right mid-upper abdomen, closely related to the hepatic flexure of the colon. Considering the patient’s age and the location, a preliminary clinical diagnosis of gastrointestinal stromal tumor (GIST) is reasonable. GIST is the most common mesenchymal tumor of the digestive tract, predominantly occurring in the stomach and small intestine, with colonic GIST being relatively rare. It typically presents as a well-defined solid mass with heterogeneous enhancement on contrast scans. However, in our case postoperative pathology and immunohistochemistry results ruled out GIST (CD117, DOG1 negative), pointing instead to mesothelioma (5, 6). Differential diagnosis should focus on excluding the following tumors: 1)Metastatic adenocarcinoma: Immunohistochemistry (e.g., CK20, CDX-2 negative) is needed to exclude metastatic colon adenocarcinoma. 2)Soft tissue sarcoma: Negative markers such as S-100, Myo-D1, myoglobin, Caldesmon, and SMA rule out neurogenic or myogenic tumors. 3)Germ cell tumors or renal clear cell carcinoma: Negative PAX-8 and renal markers exclude tumors of renal or Müllerian origin (7, 8). 4)Renal angiomyolipoma: negative markers such as HMB45 and Melan-A can be excluded.

The pathological morphology and immunohistochemical characteristics and significance are as follows: Microscopically, the tumor exhibits an adenoid and solid structure predominantly composed of epithelioid cells, with clear cytoplasm, moderate cell atypia, accompanied by extensive neoplastic necrosis, suggesting malignant biological behavior. In IHC, Calretinin, WT-1, HBME1, CK5/6 and BAP1 are important markers for the diagnosis of mesothelioma: Calretinin (nuclear/cytoplasmic positive): A specific marker for mesothelioma, used to differentiate it from adenocarcinoma (usually negative); WT-1 weakly positive: Most mesotheliomas express WT-1, and some cases are weakly positive or negative (1, 4, 10); HBME-1 was positive, which was common in mesothelioma; Although positive expression of CK5/6 is common in mesothelioma, negative expression has also been reported in a small number of cases (3). The combination of other mesothelial markers and malignant histological morphology can also be used for the diagnosis of mesothelioma; CK positive: Supports epithelial differentiation, but other markers are needed to exclude carcinoma; D2–40 partially positive: Indicates lymphatic endothelial differentiation, commonly seen in mesothelioma; Desmin is often positively expressed in benign mesothelial cells, whereas mesothelioma is relatively rare, with 10% reported in the literature (9, 10). Loss of BAP1 expression: BAP1 is mostly negative in mesothelioma, and some authors have shown that the sensitivity of BAP1 staining is about 50% (11). In this case, the tumor was located in the peritoneal cavity, and no other primary cancer metastasis was found by systemic examination. IHC showed CK, Calretinin, Desmin positive, D2-40、HBME-1 partial positive, WT-1 weak positive, BAP1 negative, and the diagnosis of mesothelioma was established.

Genetic testing has identified mutations in the ALK, MSH2, and ATM genes. Although the significance of these mutations is currently unclear, they may still hold potential clinical value. ALK Mutation: Rare in mesothelioma, but some studies suggest that ALK rearrangements may be associated with targeted therapies (e.g., crizotinib); MSH2 Mutation: May be linked to DNA mismatch repair defects, but the microsatellite instability-low (MSI-L) status in this case suggests limited benefit from immunotherapy (e.g., PD-1 inhibitors); ATM Mutation: Potentially associated with genomic instability and sensitivity to radiotherapy, though further validation is required. These genetic variants suggest that the tumor may have unique molecular driving mechanisms. However, their clinical significance needs to be clarified through functional studies or large-scale data analysis (12, 13).

The tumor in this case is a localized lesion (7.0×4.5×4.0 cm), and complete surgical resection (R0 resection) is the cornerstone of treatment. The prognosis of peritoneal mesothelioma is closely related to its histological subtype and stage: Localized epithelioid mesothelioma: Compared to diffuse malignant mesothelioma, it has a better prognosis, with a 5-year survival rate exceeding 50%; Clear cell subtype: There is limited literature on this subtype, and its biological behavior may fall between benign and malignant, necessitating long-term follow-up. In this case, no adjuvant radiotherapy or chemotherapy was administered postoperatively, possibly due to the localized nature of the tumor, complete resection, and the lack of clear guidelines for adjuvant therapy. Currently, there is no evidence supporting the survival benefit of adjuvant chemotherapy (such as pemetrexed/cisplatin regimen) after surgery for peritoneal mesothelioma. However, close monitoring for recurrence is essential (12–14).

Localized peritoneal mesothelioma is prone to misdiagnosis as gastrointestinal stromal tumor (GIST) or metastatic carcinoma. A comprehensive evaluation that integrates immunohistochemistry and clinical context is essential for an accurate diagnosis. Exploration of Molecular Characteristics:The potential of gene mutations, such as ALK, as therapeutic targets warrant further investigation, particularly for recurrent or metastatic cases. Long-term monitoring with imaging techniques (e.g., CT) and serum markers (e.g., Calretinin) is recommended. Although no recurrence was observed in this case during the 24-month follow-up, the risk of delayed metastasis in mesothelioma remains a concern (15).

Limitations: this study is a single case report with limited sample size, and the clinical significance of related molecular mutations needs to be verified by large sample studies. The follow-up time is short, and the risk of long-term recurrence and metastasis still needs further follow-up.

In Conclusion, this case, through multidisciplinary collaboration (imaging, pathology, surgery), confirmed the diagnosis of a rare localized epithelioid clear cell subtype of peritoneal mesothelioma. It highlights the curative potential of surgical resection in localized lesions. The molecular variations identified via genetic testing offer clues for future personalized treatment, though further research is needed for validation. Accumulating such cases will contribute to refining the diagnostic and treatment protocols for peritoneal mesothelioma.

## Data Availability

The data generated in the present study may be requested from the corresponding author.
